# Protective mechanical ventilation with optimal PEEP during RARP improves oxygenation and pulmonary indexes

**DOI:** 10.1186/s13063-021-05310-9

**Published:** 2021-05-19

**Authors:** Jianwei Zhou, Chuanguang Wang, Ran Lv, Na Liu, Yan Huang, Wu Wang, Lina Yu, Junran Xie

**Affiliations:** 1 Department of Anesthesia, Lishui Hospital, School of Medicine, Zhejiang University, kuocang Road 289, Lishui, 323000 Zhejiang China; 2 Department of Anesthesia, Sir Run Run Shaw Hospital, School of Medicine, Zhejiang University, East Qingchun Road 3, Hangzhou, 310016 Zhejiang China; 3 Department of Anesthesia, the Second Affiliated Hospital, School of Medicine, Zhejiang University, Hangzhou, 310009 Zhejiang China

**Keywords:** Positive-pressure respiration, Postoperative complications, Respiration, Artificial, Robotic surgical procedures

## Abstract

**Background:**

This trial aimed to evaluate the effects of a protective ventilation strategy on oxygenation/pulmonary indexes in patients undergoing robot-assisted radical prostatectomy (RARP) in the steep Trendelenburg position.

**Methods:**

In phase 1, the most optimal positive end-expiratory pressure (PEEP) was determined in 25 patients at 11 cmH_2_O. In phase 2, 64 patients were randomized to the traditional ventilation group with tidal volume (VT) of 9 ml/kg of predicted body weight (PBW) and the protective ventilation group with VT of 7 ml/kg of PBW with optimal PEEP and recruitment maneuvers (RMs). The primary endpoint was the intraoperative and postoperative PaO_2_/FiO_2_. The secondary endpoints were the PaCO_2_, SpO_2_, modified clinical pulmonary infection score (mCPIS), and the rate of complications in the postoperative period.

**Results:**

Compared with controls, PaO_2_/FiO_2_ in the protective group increased after the second RM (*P*=0.018), and the difference remained until postoperative day 3 (*P*=0.043). PaCO_2_ showed transient accumulation in the protective group after the first RM (T2), but this phenomenon disappeared with time. SpO_2_ in the protective group was significantly higher during the first three postoperative days. Lung compliance was significantly improved after the second RM in the protective group (*P*=0.025). The mCPIS was lower in the protective group on postoperative day 3 (0.59 (1.09) vs. 1.46 (1.27), *P*=0.010).

**Conclusion:**

A protective ventilation strategy with lower VT combined with optimal PEEP and RMs could improve oxygenation and reduce mCPIS in patients undergoing RARP.

**Trial registration:**

ChiCTR ChiCTR1800015626. Registered on 12 April 2018.

**Supplementary Information:**

The online version contains supplementary material available at 10.1186/s13063-021-05310-9.

## Background

Prostate cancer is the most common cancer in men, with approximately 1,414,259 new cases and 375,304 deaths recorded in 2020 [1]. The incidence of prostate cancer is rapidly growing in Asians because of a Westernized lifestyle and improved life expectancy [2]. Prostatectomy can achieve a curative effect in patients with prostate cancer [3], but it is a challenging operation because of the narrow confines of the pelvis, increasing the risk of positive surgical margins [4–6].

Hence, robotic techniques, particularly robot-assisted radical prostatectomy (RARP), are well-received in urological surgery due to operative precision in the confined pelvic space [7, 8]. During the RARP operation, the patients are placed in the steep Trendelenburg position (20°–25°) because of robot setup requirements. This position, combined with carbon dioxide (CO_2_) pneumoperitoneum at 1.6–2 kPa, leads to an upward movement of the diaphragm, alveolar collapse, and decreased pulmonary compliance and functional residual capacity [9]. Therefore, there is an increased risk of intraoperative hypoxia and postoperative respiratory complications. In addition, most prostate cancer patients are aged and need prolonged mechanical ventilation because of the time-consuming RARP surgery, which has been defined as a risk factor for pulmonary injury [10]. Overall, perioperative anesthesia management during RARP is a challenge in respiratory care.

Currently, the mechanical ventilation strategy of lower tidal volume (VT) associated with optimal positive end-expiratory pressure (PEEP) and intermittent recruitment maneuvers (RMs) is considered to be lung-protective in some surgical procedures, not only by alleviating pulmonary over-distension but also by eliminating atelectasis [11–14]. Lower VT is usually set at 4–8 ml/kg of predicted body weight (PBW) [15]. The optimal PEEP is defined as a level associated with maximum oxygenation, best dynamic compliance, and minimal dead space while preventing lung injury and adverse hemodynamic effects [16].

Previous studies assessing protective lung ventilation were mostly performed in the context of open abdominal surgery [17–20]. Haliloglu et al. [21] showed that the lung function after RARP is less impaired when using a VT of 6 ml/kg and a 6-cmH_2_O PEEP compared with CT at 8 ml/kg and ZEEP. Lee et al. [22] observed that the optimal PEEP is 7 cmH_2_O during RARP. Other studies examined different ventilation parameters during RARP [23–26] but did not assess different VT and PEEP levels. Therefore, limited data are available about the protective effect of mechanical ventilation on postoperative pulmonary function in patients undergoing RARP.

Therefore, the aims of the present study were (1) to use a decremental PEEP trial to titrate the optimal PEEP in patients undergoing RARP; and (2) to evaluate the effects of the protective ventilation strategy on oxygenation and postoperative pulmonary complications in patients undergoing RARP. The results could help prevent surgery-related morbidity, particularly in elderly patients with decreased lung function.

## Methods

### Patients

This was an open-label randomized controlled trial of patients planned to undergo a selective RARP between July 2017 and February 2018 (phase 1: July 2017 to mid-September 2017; phase 2: late September 2017 to February 2018) at the Sir Run Run Shaw Hospital of Zhejiang University. The patients were selected according to the admission sequence.

The inclusion criteria were (1) preoperative diagnosis of prostate cancer, (2) planned to undergo selective RARP, (3) > 65 years of age, (4) ASA grade I–III, (5) body mass index (BMI) 18–30 kg/m^2^, (6) operation time > 2 h, and (7) volunteered to participate in this study and signed the informed consent form.

The exclusion criteria were (1) respiratory comorbidities (clinical evidence or history of chronic obstructive pulmonary disease, interstitial pneumopathies, asthma, or lung surgery); (2) significant cardiac dysfunction (left ventricular ejection fraction < 40%); (3) intracranial hypertension; (4) preoperative anemia (hemoglobin < 100 g/L); or (5) postoperative infection excluding pneumonia.

### Ethical approval

This study was approved by the Ethics Committee of Sir Run Run Shaw Hospital of Zhejiang University (Ref. 20170622-15) on June 22nd, 2017. All methods were performed in accordance with the relevant guidelines and regulations. It was registered at the Chinese Clinical Trials Register (#ChiCTR1800015626), the registration date is 12/04/2018.

### Study design

The study was divided into two phases. In phase 1, 25 patients undergoing RARP received a recruitment maneuver (RM) and a stepwise PEEP reduction process with steps of 2 cmH_2_O starting from 15 cmH_2_O. Pulmonary compliance, oxygenation index, dead space fraction, and P(A-a)O_2_ were compared to determine the optimal PEEP. In phase 2, 64 patients were randomly allocated to two groups, including one traditional ventilation group with VT of 9 ml/kg of PBW and the other protective ventilation group with VT of 7 ml/kg of PBW with optimal PEEP and RMs.

### Anesthetic management

All patients received general anesthesia. The patients were pretreated with midazolam 0.03 mg/kg in the holding area. After arrival in the operating room, patients were monitored with non-invasive arterial pressure, electrocardiography, pulse oximetry, and temperature. The radial artery and central venous catheters were inserted to monitor blood pressure, arterial blood gas analysis, and central venous pressure (CVP). Cardiac output (CO) and stroke volume variation (SVV) were measured through an Edwards FloTrac sensor and Vigileo monitor (Edwards Lifesciences Corporation, Irvine, CA, USA).

Induction of anesthesia was carried out after a 3-min preoxygenation with a fraction of inspired oxygen (FiO_2_) of 1.0, using intravenous sufentanil (0.3–0.6 μg/kg of PBW), propofol (1.5–2 mg/kg of PBW), and rocuronium (0.6 mg/kg of PBW). PBW was calculated according to the formula: 50 + 0.91[height (cm) − 152.4] for men [27]. After intubation using an 8-mm inner diameter endotracheal tube, anesthesia was maintained with an infusion of propofol (150–200 μg/kg/min), titrated to keep a bispectral index (BIS) value of 45–55, remifentanil (0.2–0.4 μg/kg/min), and cisatracurium (1.5 μg/kg/min) as needed to keep a train-of-four (TOF) of 0 until 30 min before the end of the surgical suture. The patients were extubated in the operating room when BIS reached 75, and TOF was > 0.9.

### Ventilation protocol

**Fig. 1:**
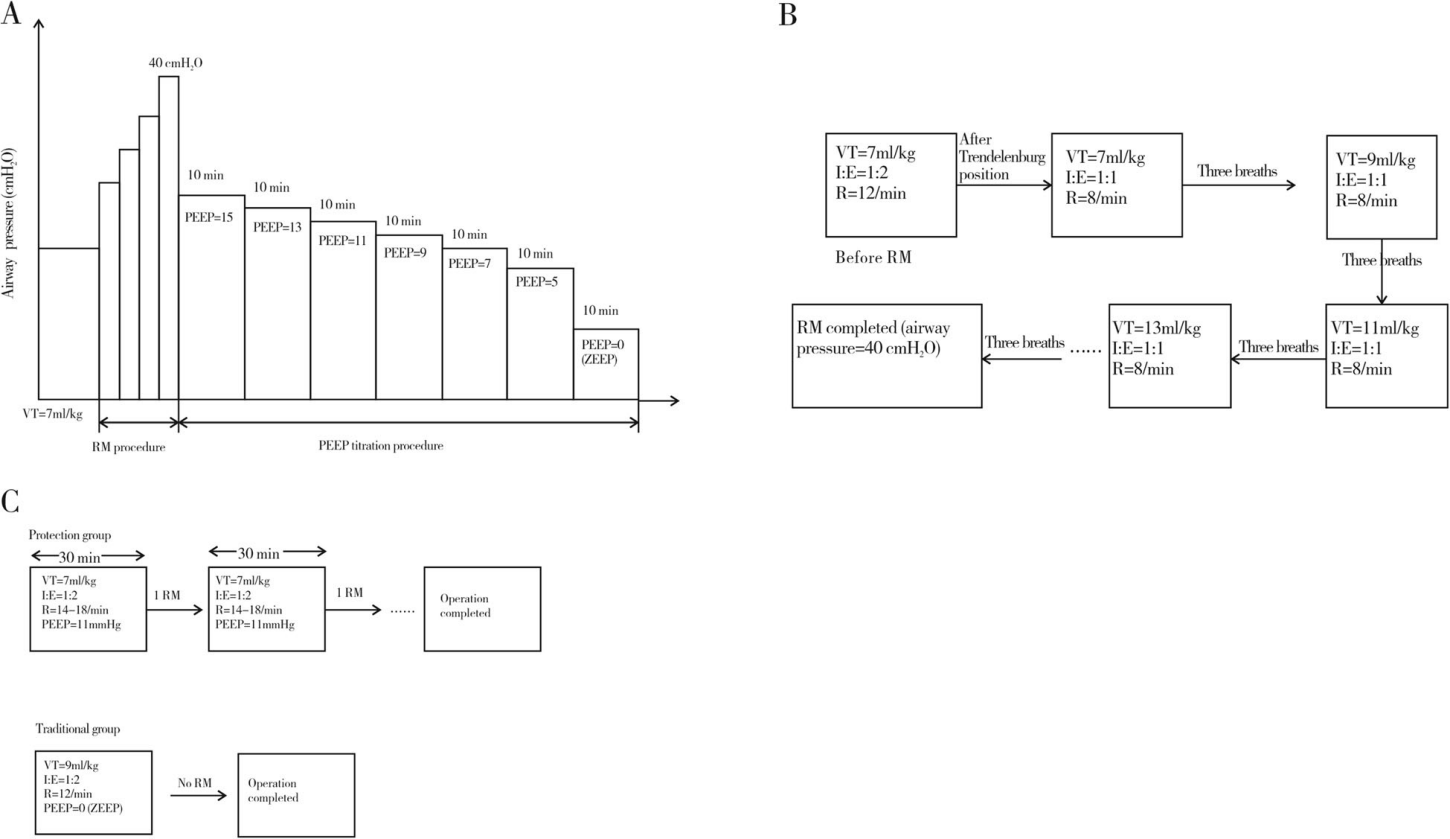
Study design. **a** Phase 1. PEEP was reduced in steps of 2 cmH_2_O from 15 to 5 cmH_2_O every 10 min and finally in one step to ZEEP. **b** RM procedure in phase 1. VT was increased from 7 ml/kg in steps of 2 ml/kg, and each step was maintained for three breaths until the target airway pressure (40 cmH_2_O) was achieved. **c** Phase 2. The traditional group was set with a VT of 9 ml/kg and the protective group with a VT of 7 ml/kg with optimal PEEP and RMs every 30 min. RM, recruitment maneuver; VT, tidal volume; PEEP, positive end-expiratory pressure; ZEEP, zero end-expiratory pressure; I, inspiratory; E, expiratory; R, respiratory rate

In phase 1, the patients were assigned to mechanical ventilation with a VT of 7 ml/kg at a FiO_2_ of 0.50, inspiratory to expiratory ratio (I:E) 1:2, and a respiratory rate of 14–18 breaths/min to maintain partial pressure of end-tidal carbon dioxide (PetCO_2_) between 4.7 and 6.0 kPa. After the patients were placed in the steep Trendelenburg position (20°–25°) and CO_2_ pneumoperitoneum (1.7 kPa) was established, RM was performed. The respiratory frequency and I:E were adjusted to 8 breaths/min and 1:1, respectively. Meanwhile, VT was increased from 7 ml/kg in steps of 2 ml/kg, and each step was maintained for three breaths until the target airway pressure (40 cmH_2_O) was achieved. Thereafter, the ventilator was switched back to the previous settings but with a PEEP of 15 cmH_2_O. PEEP was then reduced in steps of 2cmH_2_O from 15 to 5 cmH_2_O every 10 min and finally in one step to zero end-expiratory pressure (ZEEP). The ventilation protocol is depicted in Fig. 1.

In phase 2, 64 patients were randomized (using a random number table) to receive either traditional or protective ventilation. The traditional ventilation setting entailed a VT of 9 ml/kg of PBW without PEEP and RMs. The protective ventilation group was set a lower VT of 7 ml/kg of PBW with optimal PEEP added RMs every half an hour. Respiratory rates in both groups were adjusted to maintain PetCO_2_ between 4.7 and 6.0 kPa. Other respiratory parameters were the same as in phase 1.

In both phases, all patients received 10 ml/kg of Ringer’s lactate over 15 min before the induction of anesthesia. SVV was used for guiding fluid management in patients, and the value was maintained below 13%. Ephedrine and phenylephrine could be used adjunctively to keep the mean arterial pressure (MAP) within 20% of the pre-induction values. Consultant anesthetists were allowed to change the ventilation protocol at any time if there was any concern about patient safety.

### Postoperative analgesia

After induction of anesthesia, the patients received parecoxib 40 mg IV in the absence of contraindication. Tramadol 100 mg was administered IV 30 min before the end of surgery. A visual analog scale (VAS; 0, no pain; 10, worst possible pain) was used to evaluate pain intensity. Patients with VAS ≥4 could receive painkillers. Oxycodone was titrated in the recovery room. In the ward, patients were administered parecoxib 40 mg IV and an oral dose of tramadol 100 mg every 12 h.

### Perioperative observations

During surgery, heart rate (HR), MAP, CVP, CO, and SVV were collected. The variables of PetCO_2_, PEEP, peak inspiratory pressure (PIP), and VT were recorded. The dead space fraction was defined as the ratio of physiological dead space to VT (VD/VT) and was calculated as VD/VT = [arterial partial pressure of carbon dioxide (PaCO_2_) − PetCO_2_]/PaCO_2_.

In phase 1, arterial blood samples were collected at preoperation, post-induction of anesthesia (PA), post-Trendelenburg position, and pneumoperitoneum (PP), 10 min after each PEEP step, and at the end of surgical suture. The parameters were obtained from the blood gas test (Blood Gas Analyzer ABL 90 FLEX, Radiometer Medical, Copenhagen, Denmark), including arterial partial pressure of oxygen (PaO_2_), PaCO_2_, and alveolar-arterial partial pressure of oxygen difference [P(A-a)O_2_]. Dynamic compliance was measured using a spirometry system.

In phase 2, the following parameters were obtained preoperatively: pulse oximetry measured oxygen saturation (SpO_2_), arterial blood gas analysis in air, chest X-ray, and the modified Clinical Pulmonary Infection Score (mCPIS) [18]. At the post-induction of anesthesia (T1), 20 min after each RM (T2 and T3), and 30 min after extubation (T4), the following data were collected: arterial blood gas analysis and dynamic compliance (except at T4). These postoperative measurements were performed on postoperative days 1, 2, and/or 3 [28]. Past day 3, no data was collected, except hospital stay.

Modified clinical pulmonary infection score (mCPIS) calculation was made by the modified original score. The postoperative pulmonary complications (PPCs) were defined as new occurrences of three or more signs: cough, increased secretions, dyspnea, chest pain, temperature > 38 °C, and HR > 100 beats min [28]. A chest X-ray was examined in a blinded way by an independent specialist in radiology who was not involved in our study. Four pathologic features were evaluated: the increased thickness of interstitium, and disventilated areas, including minimal density change, atelectasis, and pleural effusion. Postoperative follow-up was carried out by an anesthetist who was blind to the intraoperative situation. The hospital stay was finally recorded.

### Primary and secondary endpoints

The primary endpoint of this study was the intraoperative and postoperative PaO_2_/FiO_2_. The secondary endpoints were the PaCO_2_, SpO_2_, mCPIS, and the rate of complications in the postoperative period.

### Statistical analysis

The sample size was calculated using the PASS 11.0 software (NCSS, Kaysville, UT, USA). All other statistical procedures were performed using SPSS 22.0 (IBM, Armonk, NY, USA). As a pilot study to determine the optimal mechanical ventilation parameters, the phase 1 trial had no sample size calculation and simply included 25 patients [29]. In phase 2, sample size calculation was performed as previously suggested [30]: *n* = (Z_α/2_ + Z_β_)^2^ × 2 × (standard deviation)^2^/(μ_1_ − μ_2_)^2^, where *n* is the sample size required in each group; μ_1_ and μ_2_ are mean PaO_2_/FiO_2_ in the protective and traditional ventilation groups, respectively; clinically, μ_1_ − μ_2_ = 30 mmHg; Z_α/2_ reflects a 5% level of significance (1.96); Z_β_ indicates an 80% power (0.84); standard deviation was 40 mmHg. Therefore, an *n* of 28 for each group was obtained. Considering a loss to follow-up rate of 10%, a sample size of 31 per group was adopted. The normality of the distribution was tested with the Kolmogorov-Smirnov test. Data are presented as mean (SD) or median and IQR. The data were analyzed using repeated measure ANOVA, and the post hoc pair-wise comparisons were performed using the Tukey test or using Friedman’s test (non-parametric). All tests were two-tailed, and *P* < 0.05 was considered statistically significant.

## Results

### Recruitment process

**Fig. 2:**
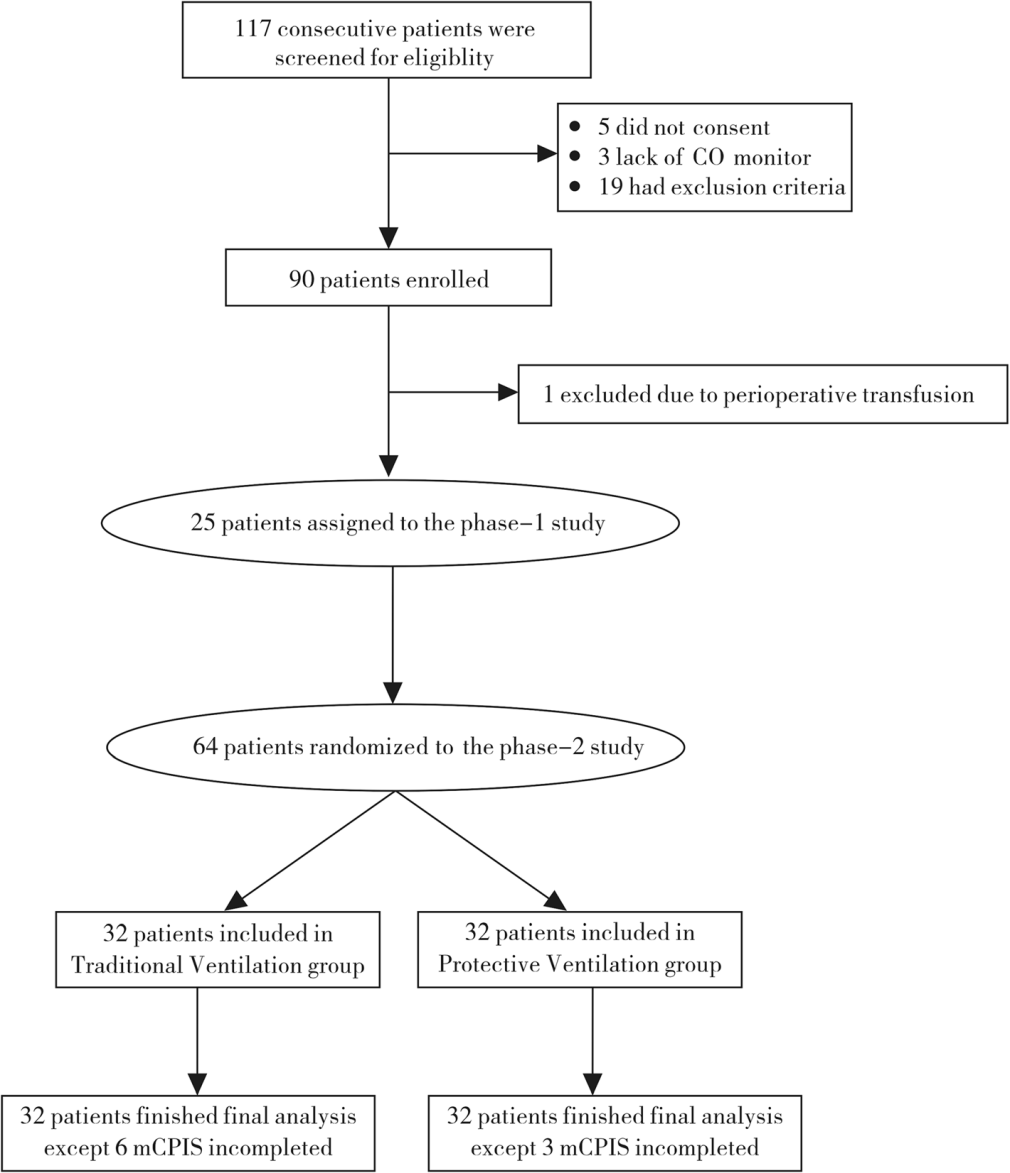
Patient flowchart. CO, cardiac output; mCPIS, modified Clinical Pulmonary Infection Score

Among 117 screened patients, 89 were finally recruited; 25 patients were assigned to the phase 1 study, and 64 were randomized in equal numbers to the two ventilation groups in the phase 2 study (Fig. 2). Except for nine subjects with incomplete mCPIS (six in the traditional group and three in the protective group; eight did not undergo X-ray because of pain, and one because the attending physician did not prescribe it), all 64 patients completed the final analysis and follow-up. Baseline characteristics are shown in Table 1.

**Table 1:** Perioperative characteristics of the patients

Variables	Phase 1 (*n*=25)	Phase 2		
		Traditional ventilation (*n*=32)	Protective ventilation (*n*=32)	*P* (within phase 2)
Age (years)	68.6±7.6	69.9±6.8	70.3±6.4	0.441
Range	60–84	60–83	60–82	
Weight (kg)	67.8±9.0	64.2±9.1	63.1±9.1	0.242
BMI (kg/m^2^)	23.5±2.7	22.6±3.1	22.5±2.6	0.274
Range	17.0–28.7	17.1–29.9	17.0–28.3	
PBW (kg)	65.2±4.5	65.1±5.0	62.8±5.6	0.233
ASA grade				
II	24 (96%)	30 (93.8%)	30 (93.8%)	0.918
III	1 (4%)	2 (6.3%)	2 (6.3%)	0.918
LVEF (%)	66.3±5.4	68.5±7.3	65.6±7.6	0.208
Hypertension	17 (68%)	18 (56.3%)	15 (46.9%)	0.280
Diabetes mellitus	7 (28%)	5 (15.6%)	4 (12.5%)	0.309
Coronary disease	0	2 (6.3%)	1 (3.1%)	0.429
Cerebral infarction	1 (4%)	2 (6.3%)	1 (3.1%)	0.828
Smoking history				0.458
Never	14 (56.0%)	23 (71.9%)	21 (65.6%)	
Former	4 (16.0%)	3 (9.4%)	4 (12.5%)	
Current	7 (28.0%)	6 (18.7%)	7 (21.9%)	
Operation time (min)	184.3±64.4	183.2±64.7	175.4±52.6	0.843
Postoperative hospital stay (days)		8.3±3.9	9.3±3.7	0.494
Estimated blood loss (ml)	138 (30–600)	89 (50–300)	100 (50–250)	0.249
Fluids (ml)	1790 (1200–3250)	1616 (1200–2800)	1605 (1100–2250)	0.314
Need for vaspressors (*n*)^a^	10 (40%)	12 (37.5%)	10 (31.3%)	0.772
Ephedrine total dose (mg)^b^	7.3±2.6	6.6±1.9	7.3±2.6	0.809
Phenylephrine total dose (ug)^c^	83.3±57.7	87.5±47.9	75.0±50.0	0.998
Duration of CO_2_ pneumoperitoneum (min)	135±58	143±56	129±43	0.686

*BMI* Body mass index, *PBW* Predicted body weight, *ASA* American Society of Anesthesiologists, *LVEF* Left ventricular ejection fraction

^a^Number of patients who received ephedrine or phenylephrine

^b^Ephedrine dose represents the mean from patients who received ephedrine

^c^Phenylephrine dose represents the mean from patients who received phenylephrine

### Phase 1

**Fig. 3:**
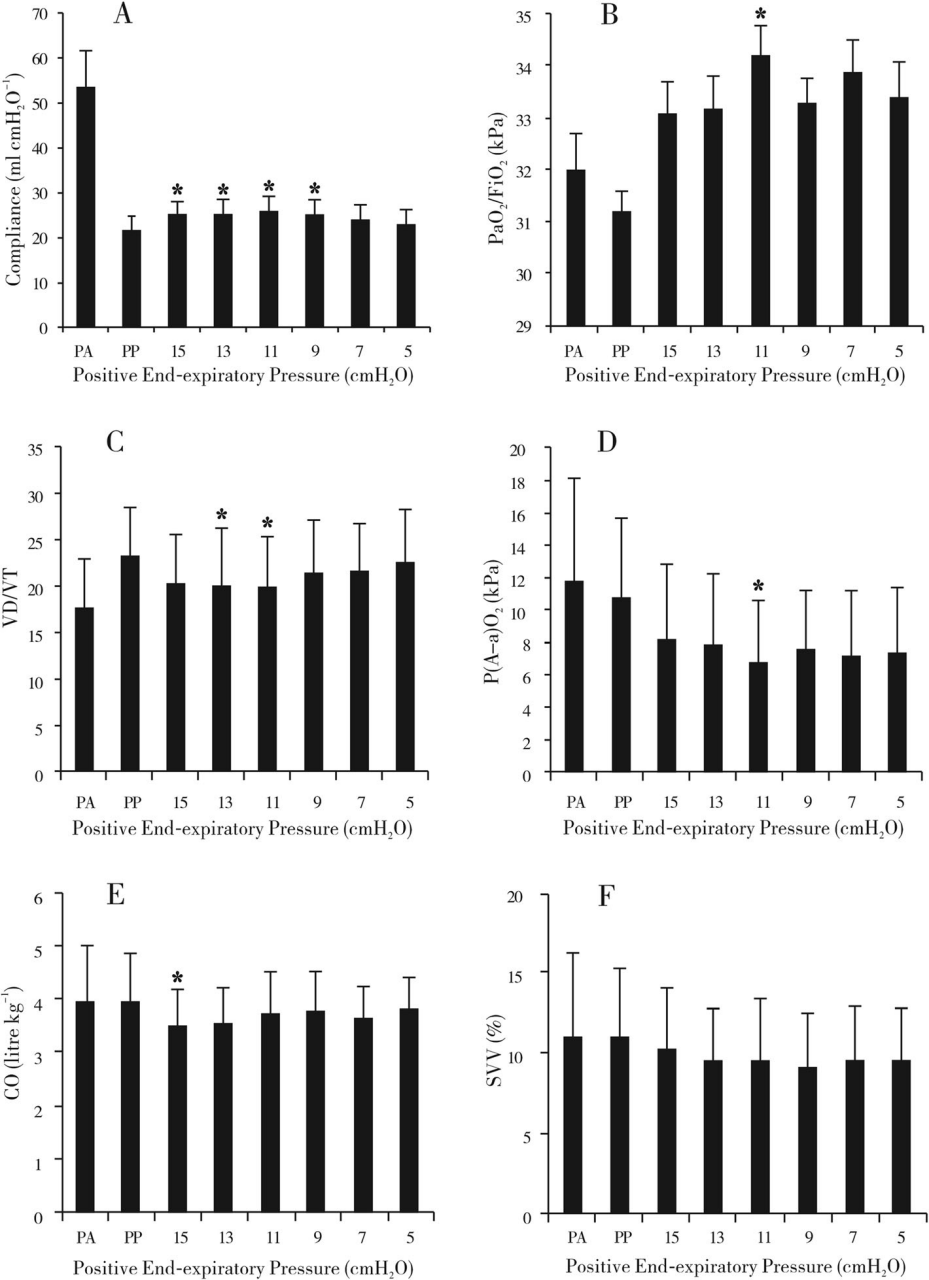
Parameters of the phase 1 study, including **a** compliance, **b** arterial partial pressure of oxygen/fraction of inspiration O_2_ (PaO_2_/FiO_2_), **c** dead space volume/tidal volume (VD/VT), **d** alveolar-arterial partial pressure of oxygen difference (P(A-a)O_2_), **e** cardiac output (CO), and **f** stroke volume variation (SVV). PA, post-induction of anesthesia; PP, pneumoperitoneum (PP); numbers on the *x*-axis, positive end-expiratory pressure (PEEP) values (cmH_2_O). *P< 0.05 versus the PP group

In the phase 1 study, a decremental PEEP titrating trial was performed to find the optimal PEEP. Compared with the time point of PP, the lung compliance increased significantly when the value of PEEP was adjusted to 15 cmH_2_O (*P*=0.014), 13 cmH_2_O (*P*=0.012), 11 cmH_2_O (*P*=0.002), or 9 cmH_2_O (*P*=0.015) with the peak compliance occurring at the PEEP level of 11 cmH_2_O. In addition, PaO_2_/FiO_2_ (*P*=0.044), VD/VT (*P*=0.042), and P(A-a)O_2_ (*P*=0.001) were also significantly improved at 11 cmH_2_O of PEEP compared with the time point of PP. With regard to CO and SVV, except that CO was decreased at the PEEP value of 15 cmH_2_O compared with the time point of PP (*P*=0.043), no obvious differences were observed at other PEEP levels (Fig. 3).

### Phase 2

**Fig. 4:**
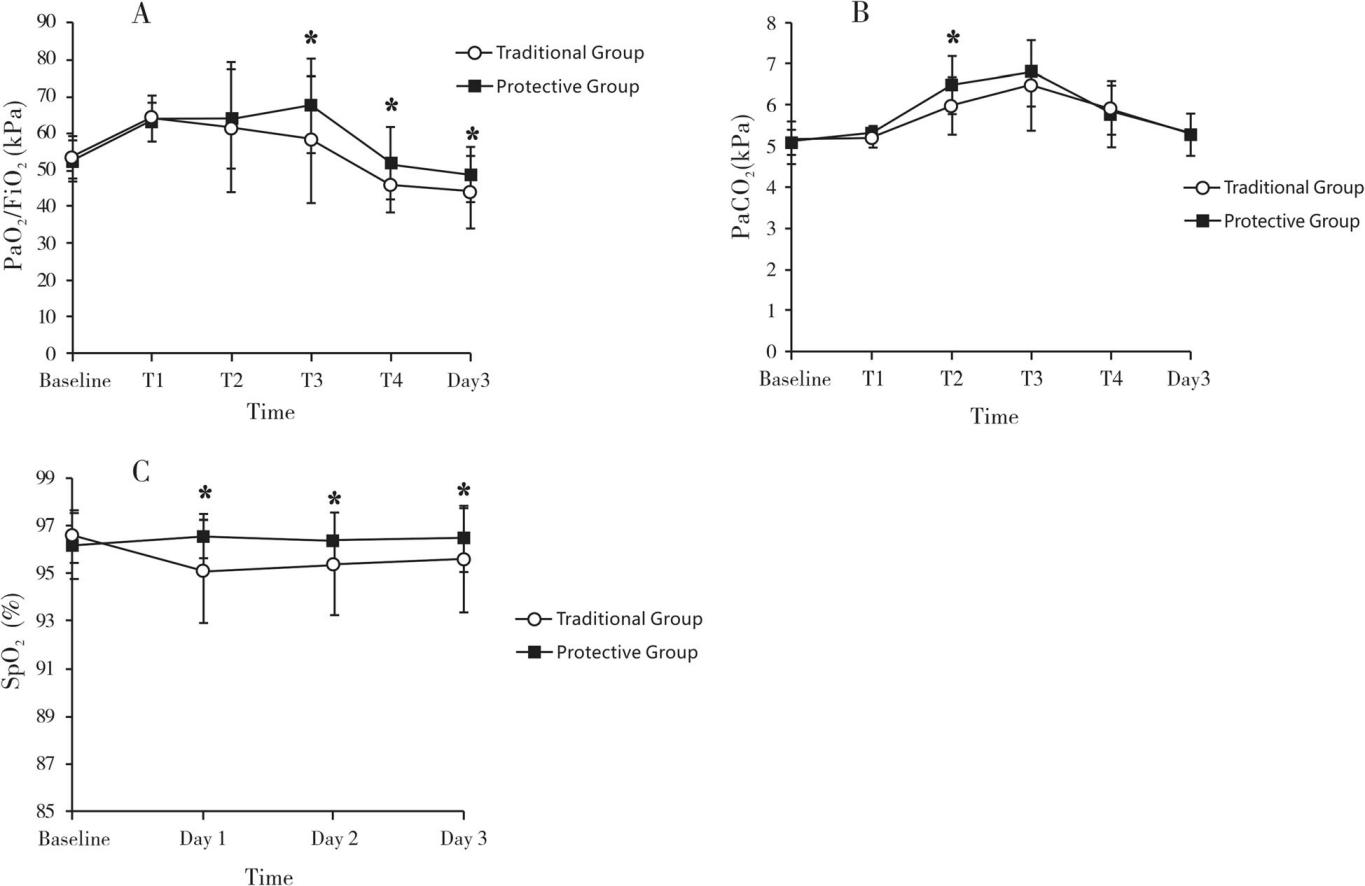
Parameters of the phase 2 study, including **a** arterial partial pressure of oxygen/fraction of inspiration O_2_ (PaO_2_/FiO_2_), **b** arterial partial pressure of carbon dioxide (PaCO_2_), and **c** pulse oximetry measured oxygen saturation (SpO_2_). Assessments were performed before anesthesia (baseline) and at post-induction of anesthesia (T1), 20 min after each RM (T2 and T3), and 30 min after extubation (T4)

In the phase 2 study, arterial blood analysis showed that compared with the traditional group, PaO_2_/FiO_2_ in the protective group apparently increased after the second RM (T3) (*P*=0.018), and the difference remained till postoperative day 3 (*P*=0.043). PaCO_2_ showed transient accumulation in the protective group after the first RM (T2), but this phenomenon disappeared with time. SpO_2_ in the protective group was significantly higher during the first three postoperative days (Fig. 4 and Supplementary Table 1).

The intraoperative hemodynamic and respiratory parameters are shown in Table 2. Compared with the traditional control, lung compliance was significantly improved after the second RM (T3) (*P*=0.025) in the protective group. Meanwhile, the plateau pressure in the protective group was seemingly higher than that in the traditional group but without statistical significance (*P*=0.051). There were no obvious hemodynamic changes with MAP, CO, and SVV in the protective group. Immediately after each RM, MAP presented transient fluctuation but remained within ±20% of the pre-induction values.

**Table 2:** Intraoperative respiratory and hemodynamic parameters during the phase 2 period

	Traditional ventilation (*n*=32)	Protective ventilation (*n*=32)	*P*
Respiratory rate (bpm)	12.8±1.5	16.3±1.3	< 0.001
Lung compliance (ml/cmH_2_O)			
T2	22.3±3.9	23.3±3.6	0.276
T3	21.4±3.7	23.5±3.7	0.025
Pplat (cmH_2_O)			
T2	26.7±3.8	28.2±1.8	0.051
T3	27.1±3.7	28.3±1.7	0.113
VD/VT			
T2	21.0±5.8	21.9±6.8	0.590
T3	22.6±5.5	21.7±6.6	0.555
MAP (kPa)			
T2	11.8±1.3	11.9±1.1	0.707
T3	11.8±1.3	12.0±1.3	0.549
CO (L/min)			
T2	3.9±0.9	3.7±0.8	0.282
T3	4.1±0.9	4.0±0.9	0.481
SVV (%)			
T2	11.2±4.0	9.8±2.7	0.098
T3	9.5±3.4	8.6±3.4	0.298

*bpm* Beats per minute, *T2 and T3* 20 min after each recruitment maneuver, *Pplat* Plateau pressure, *VD* Volume of physiological dead space, *VT* Tidal volume, *MAP* Mean arterial pressure, *CO* Cardiac output, *SVV* Stroke volume variation

The mCPIS was significantly lower in the protective group on postoperative day 3 (0.59 (1.09) vs. 1.46 (1.27), *P*=0.010) (Table 3). The two groups differed in the chest X-ray pathologic changes, with more patients showing normal X-ray at 3 days in the protective group (58.6% vs. 26.9%, *P*=0.018). Compared with the protective group, more cases in the traditional group showed patchy or diffuse infiltration on chest X-ray. The chest X-ray on postoperative day 3 demonstrated the same result that there were more severe alterations in the traditional group, including increased thickness of interstitium, disventilated areas, and atelectasis (Table 4). With regard to PPCs, although there were more postoperative cases of cough and increased secretions in the traditional group, the occurrence of PPCs was the same in the two groups (Table 5). Finally, the hospital stay was similar (traditional group: 8.3±3.9 days, protective group: 9.0±3.7 days).

**Table 3:** Comparison of the modified Clinical Pulmonary Infection Score (mCPIS) between the two groups of traditional ventilation and protective ventilation on preoperative day 0 and postoperative day 3

Components	Day 0			Day 3		
	Traditional ventilation (*n*=32)	Protective ventilation (*n*=32)	*P*	Traditional ventilation (*n*=26)	Protective ventilation (*n*=29)	*P*
Temperature (°C)						
36.1–38.4	32 (100%)	32 (100%)	> 0.99	26 (100%)	29 (100%)	> 0.99
38.5–38.9	0	0	–	0	0	–
≥39.0 and ≤36.0	0	0	–	0	0	–
Blood leukocytes (/μl)						
≥4000 and ≤11,000	31 (96.9%)	32 (100%)	> 0.99	20 (76.9%)	26 (89.7%)	0.363
< 4000 and > 11,000	1 (3.1%)	0	> 0.99	6 (23.1%)	3 (10.3%)	0.363
Tracheal secretion						
Few	30 (93.8%)	26 (81.3%)	0.257	17 (65.4%)	25 (86.2%)	0.07
Moderate	2 (6.2%)	6 (18.7%)	0.257	8 (30.8%)	4 (13.8%)	0.128
Large	0	0	–	1 (3.8%)	0	> 0.99
Purulent	0	0	–	0	0	–
PaO_2_/FiO_2_ ratio (kPa)						
> 240 or presence of ARDS	32 (100%)	32 (100%)	> 0.99	22 (84.6%)	28 (96.6%)	0.286
≤240 and absence of ARDS	0	0	–	4 (15.4%)	1 (3.4%)	0.286
Chest X-ray						
No infiltrate	28 (87.5%)	26 (81.3%)	0.491	13 (50.0%)	22 (75.9%)	0.047
Patchy or diffuse infiltrate	4 (12.5%)	6 (18.7%)	0.491	12 (46.2%)	6 (20.7%)	0.044
Localized infiltrate	0	0	–	1 (3.8%)	1 (3.4%)	> 0.99
mCPIS	0.27 (0.67)	0.41 (0.73)	0.449	1.46 (1.27)	0.59 (1.09)	0.010

*PaO*_*2*_ Arterial partial pressure of oxygen, *FiO*_*2*_ Fraction of inspired oxygen, *ARDS* Acute respiratory distress syndrome, *mCPIS* Modified clinical pulmonary infection score

**Table 4:** Results of chest X-ray test in the two groups of traditional ventilation and protective ventilation on preoperative day 0 and day 3

	Day 0			Day 3		
	Traditional ventilation (*n*=32)	Protective ventilation (*n*=32)	*P*	Traditional ventilation (*n*=26)	Protective ventilation (*n*=29)	*P*
Normal	27 (84.4%)	26 (81.3%)	0.740	7 (26.9%)	17 (58.6%)	0.018
Increased thickness of interstitium	3 (9.4%)	4 (12.5%)	> 0.99	10 (38.5%)	7 (24.1%)	0.251
Disventilatory areas including minimal density change	2 (6.2%)	2 (6.2%)	> 0.99	6 (23.1%)	5 (17.3%)	0.589
Atelectasis	0 (0%)	0	–	3 (11.5%)	0	0.198
Pleural effusions	0 (0%)	0	–	0	0	–

**Table 5:** Comparison of postoperative pulmonary complications (PPCs) between the two groups of traditional ventilation and protective ventilation on postoperative days 0, 1, 2, and 3

Complications	Day 0			Day 1			Day 2			Day 3		
	Traditional ventilation (*n*=32)	Protective ventilation (*n*=32)	*P*	Traditional ventilation (*n*=32)	Protective ventilation (*n*=32)	*P*	Traditional ventilation (*n*=32)	Protective ventilation (*n*=32)	*P*	Traditional ventilation (*n*=32)	Protective ventilation (*n*=32)	*P*
Cough	2 (6.3%)	3 (9.4%)	0.641	16 (50.0%)	8 (25.0%)	0.039^*^	18 (56.3%)	8 (25.0%)	0.011^*^	17 (53.1%)	11 (34.4%)	0.131
Increased secretions	4 (12.5%)	8 (25.0%)	0.200	20 (62.5%)	12 (37.5%)	0.046^*^	21 (65.6%)	12 (37.5%)	0.024^*^	18 (56.3%)	12 (37.5%)	0.133
Dyspnea	0	0	–	1 (3.1%)	0	>0.99	1 (3.1%)	0	>0.99	1 (3.1%)	0	>0.99
Chest pain	0	0	–	0	0	–	0	0	–	0	0	–
Temperature >38 °C	0	0	–	5 (15.6%)	1 (3.1%)	0.086	3 (9.4%)	0	0.237	1 (3.1%)	0	>0.99
HR >100 bpm	0	0	–	1 (3.1%)	0	>0.99	2 (6.3%)	0	0.472	1 (3.1%)	0	>0.99
Occurrence of PPCs	0	0	–	0	1 (3.1%)	>0.99	2 (6.3%)	0	0.472	1 (3.1%)	0	>0.99

*HR* Heart rate, *bpm* Beats per minute, *PPCs* Postoperative pulmonary complications

*P< 0.05 Comparison between the two groups was significant difference

## Discussion

The steep Trendelenburg position and capnoperitoneum during RARP may impair the postoperative lung function. This trial aimed to evaluate the effects of a protective ventilation strategy on oxygenation and respiratory complications in patients undergoing RARP. In the first phase, a PEEP reduction trial was performed during RARP to identify the optimal PEEP of 11 cmH_2_O. In the second phase, compared to the traditional ventilation with high VT, the lung-protective strategy with lower VT, optimal PEEP, and RMs were shown to improve intraoperative and postoperative oxygenation and decrease mCPIS on postoperative day 3.

In most studies about intraoperative protective mechanical ventilation, the level of PEEP applied was not individualized, and arbitrarily selecting a level of PEEP in different patient populations and surgical procedures would contribute to the heterogeneity of results [31]. Certainly, the level of PEEP should be selected according to the patient’s particular characteristics, the particularities of the surgical approach, and patient positioning [32, 33]. Until now, various indicators can be used to identify an optimal PEEP, including oxygen partial pressure, dead space fraction, lung dynamic compliance, ventilation pattern measured by computed tomography (CT), and an inflection point on the pressure/volume curve (P/V) [13, 34–40]. Maisch et al. [13, 23–26] combined best pulmonary compliance, highest oxygen partial pressure, and lowest VD/VT to determine the best PEEP of 10 cmH_2_O in faciomaxillary surgery with healthy lungs. Therefore, in the present study, we used multiple variables together to find the optimal PEEP, including oxygen partial pressure, dead space fraction, P(A-a)O_2_, and lung dynamic compliances.

In the first phase, we found that the optimal PEEP during RARP was 11 cmH_2_O. Dynamic compliance was continuously monitored during mechanical ventilation and reached the peak value when the PEEP was set at 11 cmH_2_O. Moreover, the oxygenation index and P(A-a)O_2_ were the best at PEEP of 11 cmH_2_O. Despite that the values of VD/VT were significantly decreased at PEEPs of 13 and 11 cmH_2_O, 11 cmH_2_O was finally selected as an ideal PEEP based on a cautionary principle. A high level of PEEP (> 5 cmH_2_O) would cause a higher plateau pressure and more severe lung damage, but lower VT and better compliance in the protective ventilation could help neutralize the plateau pressure and decrease the risk of lung barotrauma. Nevertheless, there was no significant difference in the plateau pressure between the two groups during phase 2. Amato et al. [41] used a multilevel mediation analysis and unclosed driving pressure (ΔP=VT/respiratory system compliance), not PEEP or VT, was an independent variable strongly associated with survival in patients with acute respiratory distress syndrome (ARDS) and only if PEEP and VT were among the changes that led to reductions in ΔP [41–43]. Alternatively, the protective ventilation approach, in which the PEEP is individualized, could result in favorable physiological effects when used intraoperatively [33, 44]. This is supported by Haliloglu et al. [21], who showed that the lung function after RARP was less impaired when using VT of 6 ml/kg and 6-cmH_2_O PEEP compared with CT of 8 ml/kg and ZEEP, and by Lee et al. [22], who observed that the optimal PEEP was 7 cmH_2_O during RARP.

It is demonstrated that PEEP is the most effective for optimizing lung function when an RM is performed before the application of PEEP [10, 11, 45]. RM can be performed in different ventilator ways, but most types of published RM are derived from the two basic maneuvers: a) sustained inflation maneuver and b) cycling maneuver. For example, “bag squeezing” with 40 cmH_2_O for 40 seconds is a classical sustained inflation maneuver. Stepwise increase of VT with a constant or stepwise increase of PEEP at constant driving pressure of 15–20 cmH_2_O in pressure-controlled ventilation is the strategy of cycling maneuver. Considering that a sudden increase in inspiratory pressure and flow could cause high shear stress and hemodynamic instability, we chose the cycling maneuver rather than sustained inflation. When RM and high PEEP were applied, hemodynamic instability is usually an on-off problem hard to be predicted or avoided, but we did not observe any hemodynamic deterioration in the protective ventilation group. The surgery position and pneumoperitoneum increased CVP and promoted the venous return to the heart, which could counteract the increased intrathoracic pressure on hemodynamics. The doses of vasopressors were not different between the two groups, and vasopressor therapy was performed mostly after induction of anesthesia and position return.

Although PPCs were a secondary endpoint and were not included in the power analysis, the comparison of mCPIS showed that the occurrence of postoperative pulmonary infection differed between the two groups. The protective ventilation strategy with low VT, high PEEP, and RMs could improve oxygenation intraoperatively [11, 33, 46], but it is still controversial whether PEEP with/without RMs is protective against PPCs. A meta-analysis in 2016 demonstrated that intraoperative low tidal ventilation in conjunction with PEEP and RMs reduced the occurrence of postoperative lung infection, atelectasis, and acute lung injury [46]. Similarly, another multicenter clinical study, the IMPROVE trial, advocated the use of higher PEEP levels for protective ventilation [17]. By contrast, the PROVHILO [16] and iPROVE [47] trials had no benefits of high PEEP in reducing PPCs. Moreover, intraoperative circulatory impairment induced by high PEEP was emphasized in the PROVHILO trial. During RARP with the special surgical position in our study, low VT in combination with high PEEP and RMs has been shown potential benefit for PPCs.

The study has some limitations. First, although we designed the phase 1 study to optimize PEEP in RARP, the method was not actually individualized but rather tailored to the specific conditions of RARP. The PEEP titration procedure takes one hour, which cannot be performed for all patients entering the operating room for logistics reasons. In addition, the patients were selected to be without pulmonary conditions, which introduced a bias that limits the generalizability of the results. Second, because of costs and ethics, a chest X-ray was used to evaluate the lung conditions in the present study. The difference of mCPIS between the two groups of traditional ventilation and protective ventilation on postoperative day 3 was mainly ascribed to the comparison of chest X-ray test. Compared to CT or MRI, chest X-ray may underestimate the presence of atelectasis and pulmonary morphology alterations [18]. A more accurate imaging technique should be applied. Third, the lung functions were assessed only for the first three postoperative days, while the mean hospitalization lasted 8–9 days. In addition, no follow-up lung function testing was performed. Finally, patient characteristics may influence the optimal mechanical ventilation parameters, and BMI in this study had a large variation. Due to the relatively small sample size, subgroup analyses could not be properly performed.

## Conclusions

We designed a two-phase clinical trial to observe the effects of a protective ventilation strategy on oxygenation and PPCs in patients undergoing RARP. The results showed that low VT in combination with optimal PEEP (11 cmH_2_O) and RMs could improve both intraoperative and postoperative oxygenation, as well as potentially reduce the occurrence of pulmonary infection. Larger clinical trials are needed to confirm whether the protective ventilation strategy could fit other events of PPCs.

## Supplementary Information

**Additional file 1: Supplementary Table 1.** Oxygenation parameters during the phase 2 period.

## Data Availability

The datasets used and analyzed during the current study are available from the corresponding author on reasonable request.

## Abbreviations

RARP: Robot-assisted radical prostatectomy
PEEP: Positive end-expiratory pressure
VT: Tidal volume
PBW: Predicted body weight
CO2: Carbon dioxide
RMs: Recruitment maneuvers
RM: Recruitment maneuver
CVP: Central venous pressure
CO: Cardiac output
SVV: Stroke volume variation
TOF: Train-of-four
PetCO_2_: Pressure of end-tidal carbon dioxide
ZEEP: Xero end-expiratory pressure
MAP: Mean arterial pressure
PA: Post-induction of anesthesia
PP: Pneumoperitoneum
PPCs: Postoperative pulmonary complications
