# Supplementary material for: Protective mechanical ventilation with optimal PEEP during RARP improves oxygenation and pulmonary indexes
Source: Trials. 2021 May 19;22:351. doi: 10.1186/s13063-021-05310-9 (PMC8135157; doi:10.1186/s13063-021-05310-9)
Supplement: Supplementary file 1 — Additional file 1: Supplementary Table 1. Oxygenation parameters during the phase 2 period. [file 13063_2021_5310_MOESM1_ESM.docx]

**Supplementary Table 1.** Oxygenation parameters during the phase 2 period.

| Oxygenation parameters | Traditional ventilation  (n=32) | Protective ventilation  (n=32) | P |
| --- | --- | --- | --- |
| **PaO_2_/FiO_2_** | | | |
| Baseline | 402.3±42.7 | 395.1±40.9 | 0.496 |
| T1 | 481.7±47.0 | 475.5±38.5 | 0.571 |
| T2 | 462.4±132.8 | 481.1±101.9 | 0.529 |
| T3 | 437.8±130.0 | 507.1±96.4 | 0.018 |
| T4 | 345.2±52.7 | 390.1±73.6 | 0.008 |
| Day 3 | 332.0±74.1 | 366.4±55.2 | 0.043 |
| **PaCO_2_** | | | |
| Baseline | 38.3±4.0 | 38.4±2.6 | 0.903 |
| T1 | 39.4±1.3 | 39.5±1.7 | 0.746 |
| T2 | 45.1±5.4 | 48.7±5.3 | 0.008 |
| T3 | 48.6±7.9 | 51.2±5.9 | 0.149 |
| T4 | 44.4±4.7 | 43.6±6.2 | 0.583 |
| Day 3 | 39.4±3.8 | 39.8±3.4 | 0.713 |
| **SpO_2_** | | | |
| Baseline | 96.6±1.1% | 96.2±1.4% | 0.387 |
| Day1 | 95.1±2.2% | 96.6±0.9% | 0.001 |
| Day2 | 95.4±2.1% | 96.4±1.2% | 0.001 |
| Day3 | 95.6±2.2% | 96.5±1.4% | 0.030 |

T1: post-induction of anesthesia; T2 and T3: 20 min after each recruitment maneuver; T4: 30 min after extubation.
